# Poor Long-Term Efficacy of Prevnar-13 in Sickle Cell Disease Mice Is Associated with an Inability to Sustain Pneumococcal-Specific Antibody Titers

**DOI:** 10.1371/journal.pone.0149261

**Published:** 2016-02-24

**Authors:** Steven M. Szczepanek, Sean Roberts, Kara Rogers, Christina Cotte, Alexander J. Adami, Sonali J. Bracken, Sharon Salmon, Eric R. Secor, Roger S. Thrall, Biree Andemariam, Dennis W. Metzger

**Affiliations:** 1 Center of Excellence for Vaccine Research, Department of Pathobiology and Veterinary Science, University of Connecticut, Storrs, CT, United States of America, 06269; 2 Center for Immunology and Microbial Disease, Albany Medical College, Albany, NY, United States of America, 12208; 3 Department of Immunology, University of Connecticut Health Center, Farmington, CT, United States of America, 06030; 4 Helen and Harry Gray Cancer Center and Department of Medicine, Division of Integrative Medicine, Hartford Hospital, Hartford, CT, United States of America, 06106; 5 The Lea’s Foundation Center for Hematological Disorders, Neag Comprehensive Cancer Center, University of Connecticut Health Center, Farmington, CT, United States of America, 06030; Instituto Butantan, BRAZIL

## Abstract

**Background:**

One of the most common causes of morbidity and mortality in children with sickle cell disease (SCD) is infection with the pneumococcal bacterium (*Streptococcus pneumoniae*). Unfortunately, the polysaccharide-conjugate vaccine appears to be less effective in individuals with SCD when compared to the general population. We sought to better understand the relative efficacy of pneumococcal vaccination in a SCD mouse challenge model.

**Methods:**

Transgenic control and SCD mice were monitored for mortality after intranasal pneumococcal infection or pneumococcal vaccination with Prevnar-13 and type-matched challenge. Anti-pneumococcal antibody titers were measured by ELISA and opsonophagocytosis was measured *in vitro*.

**Results:**

Mortality after pneumococcal infection was similar between control and SCD mice. However, after three intramuscular polysaccharide-conjugate vaccinations, all control mice were protected following high-dose intranasal infection, whereas 60% of SCD mice died. Anti-pneumococcal antibody titers showed initial IgG and IgM responses in both groups, but waning titers were observed in the SCD group, even after boosting. When functionally assayed *in vitro*, serum from SCD mice 13 weeks after a second booster shot maintained little to no ability to opsonize pneumococci, while serum from control mice sustained a significantly higher capacity opsonization. Thus, it appears that SCD mice do not maintain antibody responses to pneumococcal polysaccharides after Prevnar-13 vaccination, thereby leaving them susceptible to mortality after type-matched infection.

**Conclusion:**

Our results emphasize the need to better understand the correlates of immune protection in SCD so that pneumococcal vaccines can be improved and mortality reduced in this susceptible population.

## Introduction

Sickle cell disease (SCD) is a complex hematological disorder that has a dramatic effect on immunity and resistance to infection. The current case-fatality rate within this population due to infection (when not adjusted for age) stands at approximately 14% [1]. Children with SCD are at a particularly high risk for infection with the encapsulated bacterium, *Streptococcus pneumoniae* (i.e. pneumococcus), which is presumably due to altered splenic architecture and function [2]. A life-threatening condition arises if the bacterium becomes invasive and causes bacteremia (known as invasive pneumococcal disease, or IPD). IPD has been found to be 10–100 times more prevalent in children with SCD than the general population [3] and is even twice as likely in individuals with sickle cell trait [4, 5]. Given the extreme morbidity and mortality associated with pneumococcal infection in the SCD population, current clinical guidelines dictate that these patients be placed on prophylactic penicillin at approximately 4 months of age and adhere to a strict regimen of pneumococcal vaccination.

Cases of splenectomy have demonstrated that the spleen is a crucially important organ in protection from IPD in both humans [6] and mouse models [7]. Previous vaccination appears to be sufficient to maintain antibody titers in many cases of splenectomy; however, retention of memory B-cells is adversely affected [8]. Furthermore, while it is agreed upon by most in the field that anti-pneumococcal titers are induced in children with SCD shortly after vaccination, it has been reported that titers may not be maintained long-term after vaccination with the un-conjugated pneumococcal polysaccharide vaccine [9], indicating that these children may have defects in the generation of memory B-cells and/or long-lived plasma cells. Protection from IPD has been demonstrated to rely heavily on the presence of “memory IgM B-cells” (human) or “B-1a B-cells” (mouse) [10, 11]. These cells produce antibodies that target carbohydrate moieties commonly found on encapsulated bacteria. The presence of a functional spleen has been shown to be essential to the survival of these cells [12]. Interestingly, we have previously shown that splenic architecture is disrupted in transgenic SCD mice and B-1a B-cells are dramatically reduced in number in the spleens of these mice [13]. Hence, it is likely that the generation of a robust plasma cell and memory B-cell response is essential to thwart recurrent pneumococcal infection, and a lack thereof may be responsible for increased susceptibility in children with SCD who lack splenic function and normal numbers of memory IgM B-cells.

Since the introduction of the use of prophylactic penicillin and the newer pneumococcal polysaccharide-protein conjugate vaccine Prevnar in children with SCD, hospitalization associated with infection from this pathogen has been reduced three-fold [14] and infection has been concomitantly reduced to approximately one-third of its previous level [15]. Unfortunately, this still leaves room for improvement in treatment and therapies to combat infection by this pathogen in children with SCD. Given the strict adherence to pneumococcal vaccination in SCD patients at many hematology clinics, this phenomenon is surprising and vaccine failure may be to blame for some of these cases. While little is known about the ability of Prevnar to specifically protect from type-matched infection in SCD patients, we do know that the 23-valent pneumococcal polysaccharide vaccine has been shown to have little to no efficacy in SCD patients in some reports, even after administering a booster dose [16, 17]. Hence, the efficacy of pneumococcal vaccination does not appear to be as high in children with SCD when compared to the general population.

Immune dysregulation in the transgenic SCD mouse model has recently become apparent. We have shown that disrupted splenic architecture is prevalent at a young age in these mice, as are aberrations in the distribution of lymphocyte populations, cytokines/chemokines, and antibody classes [13]. Further changes in immunity have been noted after animals received a vaccination with ovalbumin and the adjuvant aluminum hydroxide (OVA/alum). These vaccinations resulted in high IgE titers, further dysregulation of cytokines/chemokines/antibodies, and a notable increase in the levels of IL-1β and IL-6 in bronchoalveolar lavage fluid of the SCD mice [18]. Given our previous findings that immunity is dysregulated in the SCD mouse model, we hypothesize that immunity is impaired in SCD and drives the reduced pneumococcal vaccine efficacy that has been clinically observed in this population. Herein we describe the immunogenicity and efficacy of the pneumococcal polysaccharide-conjugate vaccine Prevnar-13 in the SCD mouse model to address the above hypothesis.

## Materials and Methods

### Animal Research Ethics Statement

This study was carried out in strict accordance with the recommendations in the Guide for the Care and Use of Laboratory Animals of the National Institutes of Health. The protocol was approved by the Institutional Animal Care and Use Committee at Albany Medical College (protocol #14–04003; Metzger) and the University of Connecticut (protocol #A14-029; Szczepanek). During experimental infections, mice were weighed daily and checked for signs of distress. Mice that were determined to exhibit moderate to severe clinical signs (i.e. severely reduced mobility or persistent recumbency) were considered for euthanasia via an overdose of the anesthetic sodium pentobarbital followed by cervical dislocation (conducted by staff trained in the use of the technique).

### Mice

Two to three month-old female mice harboring knock-ins of the human alpha globin transgene, along with either a normal human beta globin or sickle beta globin transgene [B6;129-*Hba*^*tm1(HBA)Tow*^ *Hbb*^*tm2(HBG1*,HBB^*^)Tow^/*Hbb*^*tm3(HBG1*,*HBB)Tow*^/J; stock number 013071] were purchased from Jackson Laboratories (Bar Harbor, ME). Mice homozygous for the normal human beta globin transgene are henceforth referred to as “control” mice and those homozygous for the sickle beta globin transgene are referred to as SCD mice. Serum for ELISA was obtained via retro-orbital bleed. Five to eight mice were used for all experiments. All mice were maintained and utilized at the Albany Medical College Animal Facility.

### Bacteria

The A66.1 *S*. *pneumoniae* strain, which expresses pneumococcal polysaccharide serotype 3 (PPS3), was cultured at 37°C in Todd-Hewitt broth until mid-log phase (using BSL-2 precautions), washed and resuspended in fresh broth containing 15% glycerol, and stored at −70°C until use.

### Infection

Infection was induced in naïve, unvaccinated mice intraparitoneally anesthetized with ketamine HCl (Fort Dodge Animal Health, Fort Dodge, IA) and xylazine (Phoenix Scientific, St. Joseph, MO) in PBS and inoculated intranasally with a dose of 5 X10^4^ CFU of A66.1 pneumococci in 50 μl of Ringer's solution. Mice were weighed daily, monitored for signs of distress or illness, and mortality was recorded. Lungs were removed from mice that died during infection and were fixed overnight in 10% formalin, embedded in paraffin and sectioned, and H+E stained using standard protocols of the CT Veterinary Medical Diagnostic Laboratory (CVMDL) at the University of Connecticut. Resolution of *S*. *pneumoniae* in the lungs was performed using a Gram stain of paraffin-embedded tissues. Histologic evaluation of lung lesions was conducted by a board-certified veterinary pathologist from the CVMDL.

### Vaccination and Challenge

**Fig 1:**
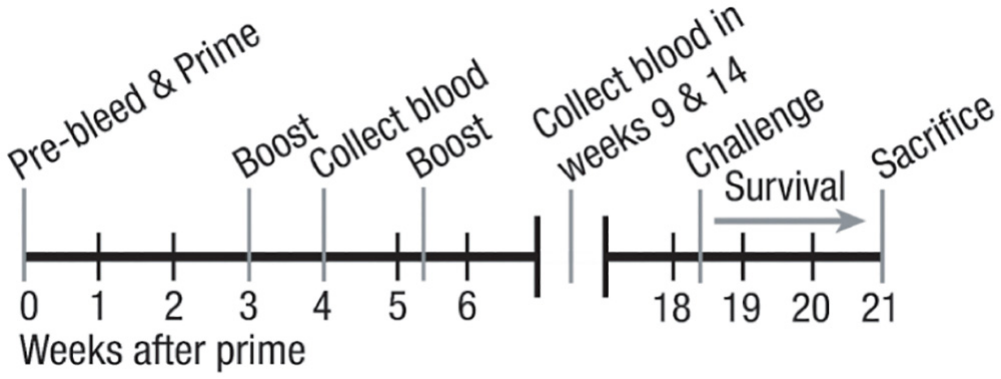
Mouse model of pneumococcal vaccination and infectious challenge. 2–3 month old mice were bled, and serum was collected prior to initial priming with Prevnar-13 on week 0. Mice were boosted on weeks 3 and 5 with the same vaccine. Mice were again bled on weeks 4, 9, and 14. Mice were then challenged with the virulent A66.1 strain of *S*. *pneumoniae* on week 18 and mortality was assessed for 16 days after challenge.

Mice were intramuscularly inoculated in the hindlimbs with Prevnar-13 (Wyeth Pharmaceuticals, Collegeville, PA) with a dose containing 0.22 μg of each polysaccharide. This vaccine includes PPS3. The mice were boosted twice, tested for serum anti-PPS3 antibodies, and challenged according to the schedule outlined in Fig 1. Challenge of vaccinated mice was conducted via intranasal inoculation of 1X10^6^ CFU of A66.1 pneumococci in 50 μl of Ringer’s solution. Mice were weighed, monitored for signs of distress or illness, and mortality was recorded.

### ELISA

PPS3-specific antibody titers were measured using ELISA. 96 well Polysorp Nunc-Immuno plates (Nalge Nunc International, Rochester, NY) were coated with 50 μL of PPS3 antigen (2 μg/mL, ATCC, Manassas, VA) and incubated at 4°C overnight. Plates were then washed three times with PBS (Life Technologies, Carlsbad, CA) containing 0.05% Tween 20 (Sigma) and then blocked with 5% fetal calf serum (Hyclone Laboratories, Inc., Logan, UT) at room temperature for 1 hr. Serial dilutions of serum in blocking buffer were added to the plates, which were then incubated at room temperature for 2 hr. After five washes with PBS-0.05% Tween20, 50 μL of biotin-conjugated goat anti-mouse IgG or IgM antibody (SouthernBiotech, Birmingham, AL) was added, and the plates were incubated at room temperature for 1 hr. After six washes, 50 μL of Streptavidin-HRP (Biosource/Life Technologies, Carlsbad, CA) was added and the plates were incubated at room temperature for 30 mins. After seven washes, 50 μL of TMB Peroxidase substrate solution was added. 50 μl of 1.8 N Sulfuric acid was used to stop the reaction. Finally, the absorbance was measured at 450 nm using a microplate reader (BioTek Instruments, Inc., Winooski, VT). Dilutions corresponding to 50% maximal binding were used to calculate serum titers.

### Opsonophagocytic Killing Assay

The protocol used is adapted from Romero-Steiner et al. [19]. Mouse macrophage J774a.1 cells obtained from ATCC (Manassas, VA) were thawed and cultured according to manufacturer recommendations. J774a.1 cells were then added to 96-well round bottom plates at a concentration of 4 x10^4^ cells/well and incubated with a pneumococcal cells (A66.1) at a concentration of 2 x 10^5^cells/well, diluted from a stock of a known concentration (using CFU counts on blood agar plates). Serum was collected from Prevnar-13 vaccinated control and SCD mice 13 weeks after their third vaccination, which was then diluted 1:2 in assay buffer. Mouse complement (Rockland Antibodies and Assays, Limerick, PA) was added at 10 μl/well (890 μg). Following final incubation, a 10 μl aliquot was diluted 1:10 in Todd-Hewitt Broth and 20 μl was plated on to blood agar plates and incubated for 18 hr at 37°C. Colonies were counted and compared to a no serum control. Data are expressed as the percentage of phagocytosis over no serum control.

### Statistics

Mortality curves were assessed for statistical differences using the log-rank/Mantel-Cox test. Statistical differences in body weight over time were determined using repeated-measures ANOVA. Differences in antibody titers were determined separately at each time-point for between group comparisons using a two-tailed t-test, while within group comparisons across time points were determined by One-way ANOVA. Opsonophagocytosis between groups was compared using a two-tailed t-test. Significance was determined as *p* < 0.05. Statistics were calculated using Prism version 6 software (GraphPad, La Jolla, CA).

## Results

### Mortality and Histopathology After Pneumococcal Infection

**Fig 2:**
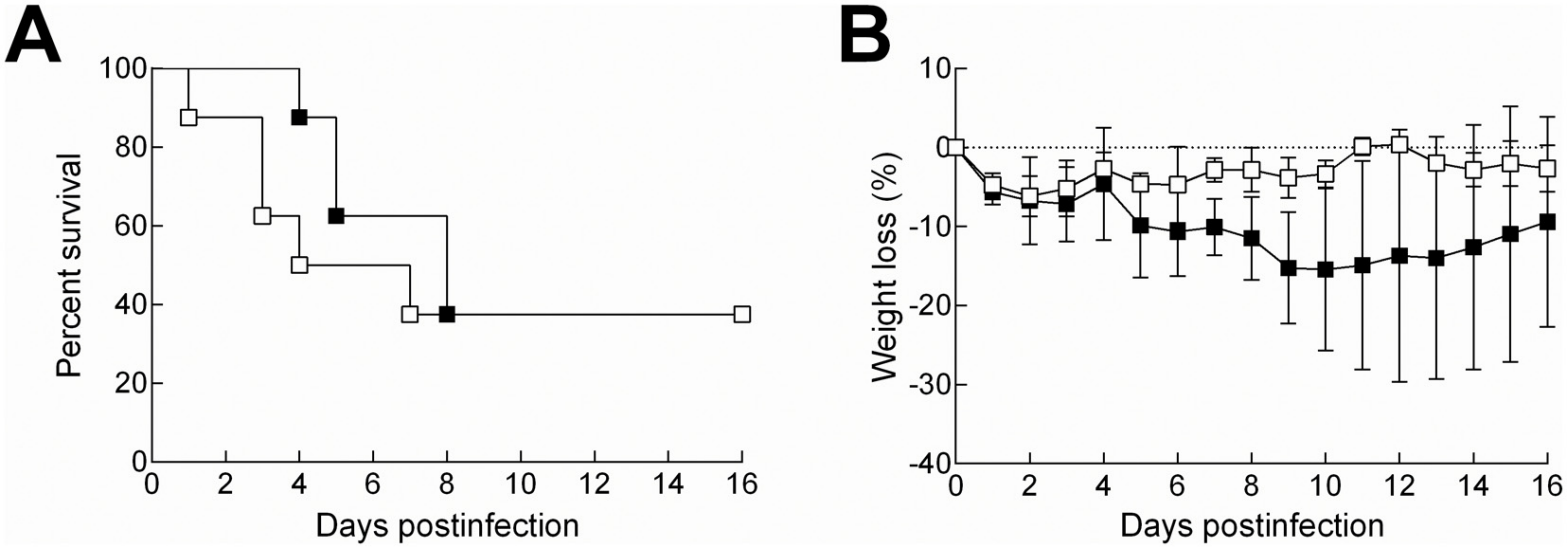
Differential mortality kinetics of control and SCD mice after infection. 2–3 month old mice were infected intranasally with 5X10^4^ CFU of A66.1 *S*. *pneumoniae* and mortality (A) and weight loss (B) were measured. No statistical differences were observed for either parameter. White boxes = SCD, black boxes = controls.

Initial pilot studies with non-transgenic wild-type mice of the same genetic background as the SCD mice identified 5X10^4^ CFU as the approximate LD_50_ for intranasal A66.1 pneumococcal infection (data not shown) and this dose was subsequently used in the SCD mouse model. Overall mortality after eight days of infection was not different in both SCD (n = 8) and control mice (n = 8). Although SCD mice tended to die earlier than control mice (Fig 2a), there was no statistical difference in the mortality curves between the two groups. Similarly, there was no statistical difference between the groups in weight lost after infection (Fig 2b).

**Fig 3:**
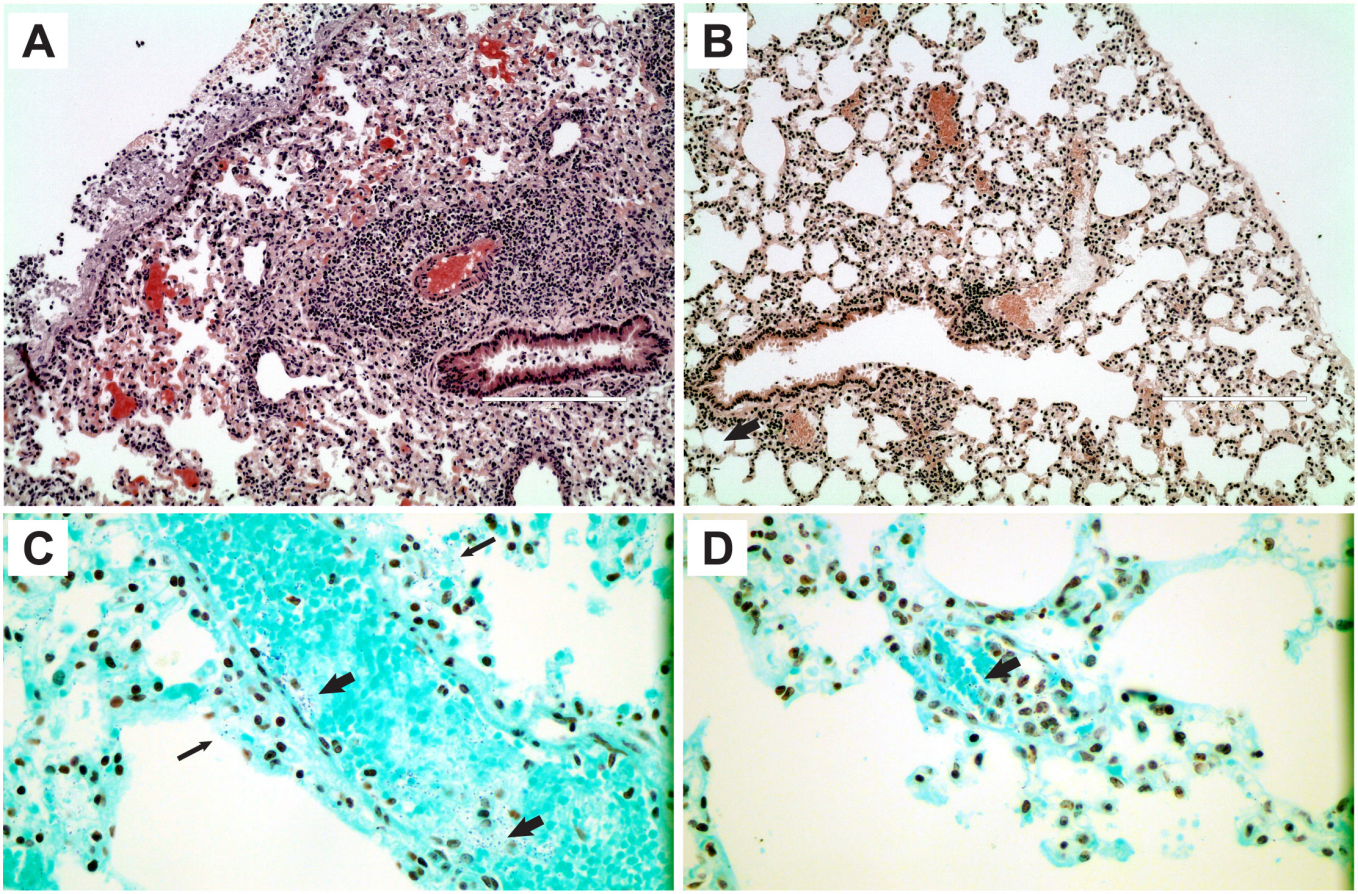
Histopathology and Gram staining of lung tissues in mice that succumb to intranasal pneumococcal infection. H+E staining of lung tissue from control (A) and SCD (B) mice after they succumbed to pneumococcal infection. Gram stain of the same lung tissue in control (C) and SCD (D) mice. Wide arrows point to pneumococci in blood vessels and narrow arrows point to pneumococci colonizing the surface of the lung tissue.

In this study, lungs from infected control mice (Fig 3a) were much more severely inflamed than those from SCD mice (Fig 3b), with notable perivascular and peribronchial infiltrates and pleuritis in the control mice that was very mild to absent in SCD mice. Pneumococcal bacteria were visually present in both the blood and attached to the surface of the lung tissue in the control mice (Fig 3c), whereas they were only observed in the blood of SCD mice (Fig 3d). Thus, lung colonization was associated with lung lesions in control mice that died from infection, but mild lung lesions were associated with the visual presence of the bacteria only in the blood of SCD mice that died from infection.

### Antigen-specific Antibody Titers and Functional *in vitro* Opsonophagocytosis of Pneumococci After Vaccination with Prevnar-13

**Fig 4:**
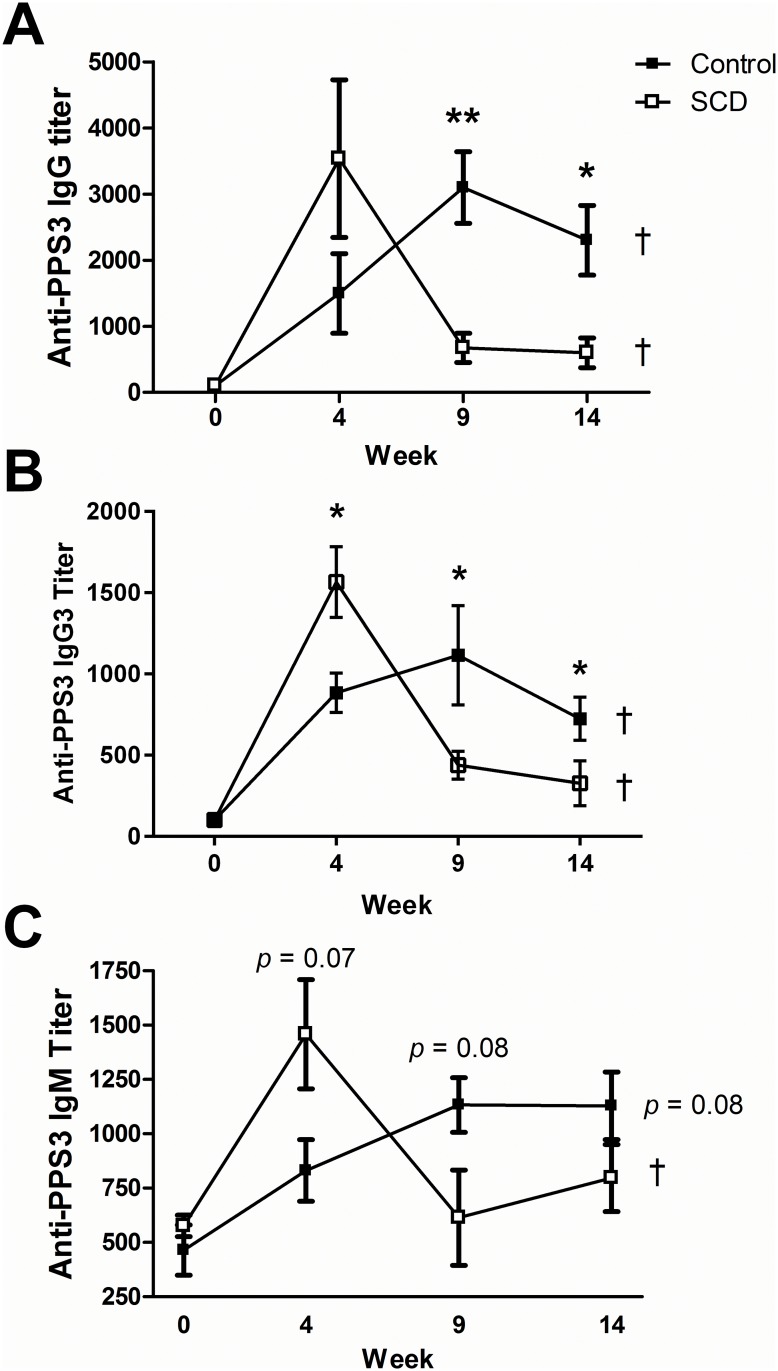
Pneumococcal-specific antibody titers after vaccination. PPS3-specific total IgG (A), IgG3 (B), and IgM (C) antibody titers were measured by ELISA in Prevnar-13 vaccinated 2–3 month old control and SCD mice at weeks 0 (before vaccination), 4, 9, and 14. * = *p* < 0.05 and ** = *p* <0.01 for between groups comparisons at the same time point (as determined using a two-tailed Student’s T-test) and † = *p* < 0.05 for within group comparisons across time points (as determined using a One-way ANOVA). For IgM comparisons between groups, *p* = 0.07 and *p* = 0.08 at weeks 4 and 9, respectively. For IgM comparisons within the control group, *p* = 0.08. White boxes = SCD, black boxes = controls.

After intramuscular priming and boosting with Prevnar-13 (as indicated in Fig 1), serum anti-PPS3 total IgG, IgG3, and IgM antibody titers were measured by ELISA. Initial anti-PPS3 total IgG and IgG3 titers increased within 4 weeks in both control (n = 5) and SCD (n = 5) mice (Fig 4), which is in keeping with clinical findings of induced antigen-specific titers shortly after vaccination in people with SCD. These titers continued to significantly increase (*p* < 0.05) in the control animals after booster shots were administered, as was expected. Conversely, anti-PPS3 total IgG and IgG3 titers significantly waned to near naïve levels in SCD mice at the 9 and 14 week time points, even with repeated boosting (*p* < 0.05). Not surprisingly then, the anti-PPS3 total IgG and IgG3 titers were significantly higher in control mice when compared to SCD mice at the 9 and 14 week time points (*p* < 0.01 and *p* < 0.05, respectively). Control mice trended towards an increasing anti-PPS3 IgM response (*p* = 0.08) over time, whereas SCD mice induced a strong initial anti-PPS3 IgM response (week 4) that again waned to near naïve levels thereafter (*p* < 0.05). These findings are in line with clinical observations that anti-pneumococcal antibody titers are not maintained long-term in people with SCD.

**Fig 5:**
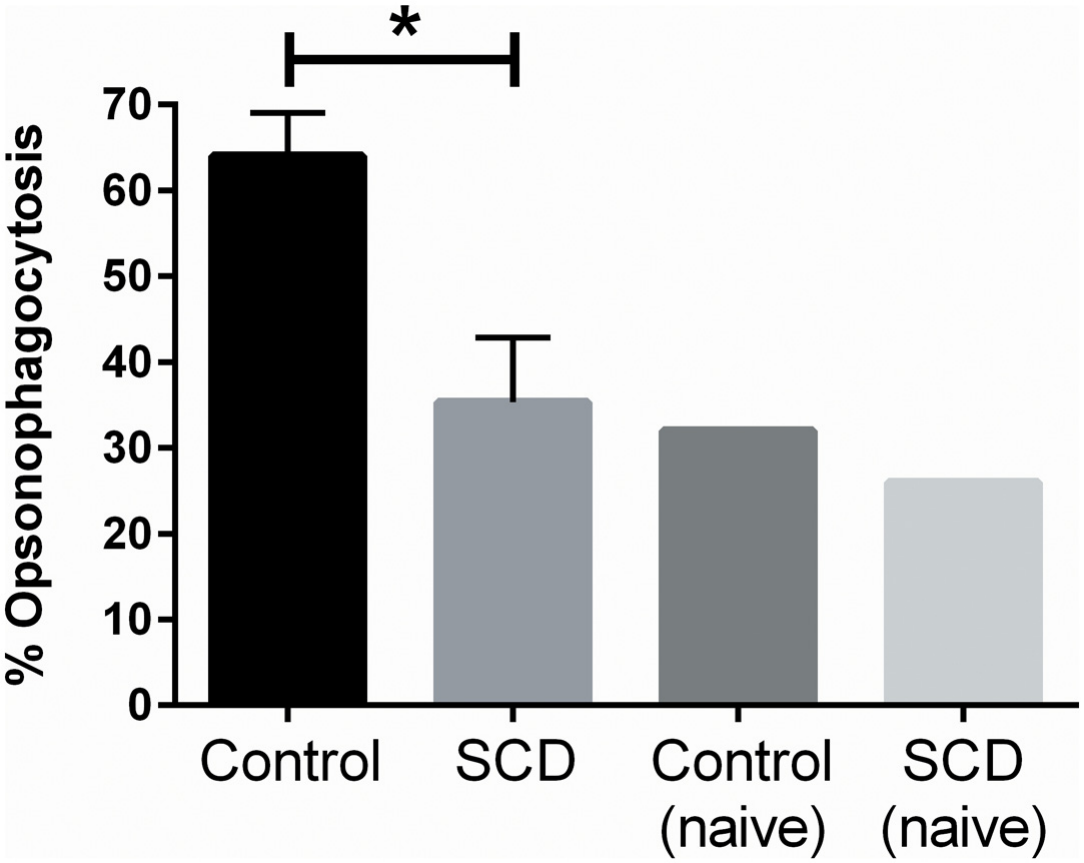
*in vitro* opsonophagocytosis of pneumococci 13 weeks after boosting with Prevnar-13. Serum from vaccinated mice (or pooled serum from naïve mice) was mixed with exogenous mouse complement and A66.1 *S*. *pneumoniae* and added to cultures of mouse macrophages *in vitro*. Mixtures were then streaked out on blood-agar plates, incubated at 37°C overnight and viable pneumococcal colonies were counted. Data are reported as the percentage of pneumococcal colonies that were not phagocytosed by macrophages when compared to “no serum control” wells. *p* < 0.05 as determined by a t-test. Comparisons to naïve mice were not performed as those samples were pooled.

Functional analysis of the long-term antibodies that are induced by three shots of Prevnar-13 were determined using an *in vitro* opsonophagocytic killing assay (Fig 5). Exogenous mouse complement was added to the assay to ensure that differences between groups for macrophages to phagocytose pneumococci were the result of differences in antibody titer and/or quality, and not due to differences in complement. Serum from control mice (n = 3) maintained a significantly higher (*p* < 0.05; mean = 64% over no serum control) ability to opsonize pneumococci than serum from SCD mice (n = 3, mean = 35% over no serum control) 13 weeks after a third shot with Prevnar-13. Notably, pooled serum from unvaccinated control and SCD mice was not much different in its capacity to opsonize pneumococci (mean = 32 and 26, respectively) than vaccinated SCD mice. These findings are in line with the nearly naïve anti-PPS3 titers observed in SCD mice after three injections with Prevnar-13 and indicate that the waning antigen-specific titers observed in SCD mice are functionally relevant.

### Mortality from Infectious Challenge After Vaccination with Prevnar-13

**Fig 6:**
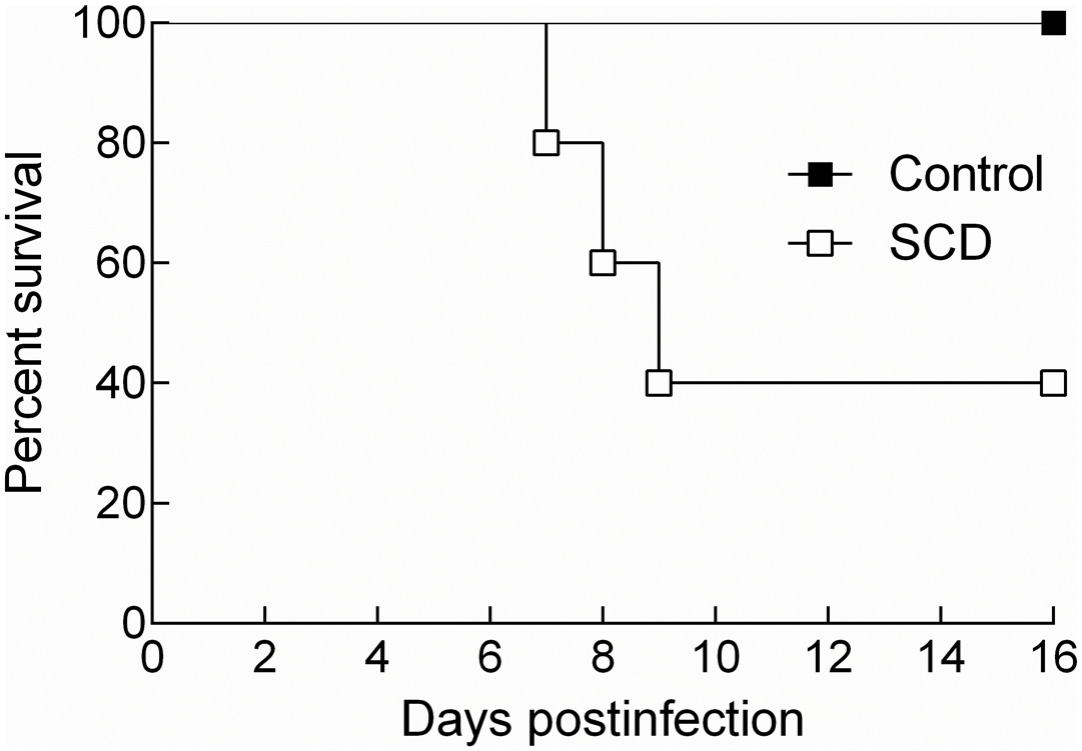
Prevnar-13 vaccination does not protect SCD mice from pneumococcal infection. Mice were vaccinated and challenged according to the schedule outlined in Fig 1. Mice were intranasally challenged with 1X10^6^ CFU of A66.1 and mortality was assessed measured. *p* = 0.01, as determined by the log-rank/Mantel-Cox test. White boxes = SCD, black boxes = controls.

We used a challenge dose of A66.1 that is approximately two orders of magnitude higher than the LD_50_ dose in unvaccinated mice to ensure that observed survival is associated with vaccination. Mice were intranasally given 1X10^6^ CFU of A66.1 pneumococci (PPS3, a serotype found in Prevnar-13) approximately three months after the last vaccine boost was administered. All control mice (n = 8) survived challenge with virulent PPS3; however, 60% of SCD mice (n = 5) died after infection (Fig 6, *p* = 0.01). Hence, the vaccine appears to have little long-term efficacy in the SCD mouse model, which correlates with the waning antibody titers observed in these animals at the time of challenge.

## Discussion

The importance of pneumococcal vaccination in children with SCD cannot be overemphasized due to the devastatingly high morbidity and mortality associated with infection by this pathogen. While the pneumococcal polysaccharide-protein conjugate vaccine Prevnar has dramatically reduced infectious burden in children with SCD, far too many cases of pneumococcal infection still occur every year in this group and little is known as to why this is the case. We found in our infection model that mortality for both control and SCD mice were similar, which was surprising given the dramatically increased vulnerability of children with SCD to pneumococcal infection. Furthermore, Miller et al. previously reported that SCD mice are hyper-susceptible to pneumococcal infection [20]. However, this study followed mice for a much shorter duration (72 hours), and our results agree with their findings up to this time point as we observed a delay in mortality in the control mice when compared to SCD mice early in infection. Interestingly, SCD mice that died from pneumococcal infection had mostly mild lung lesions, and pneumococci could only be found in the blood when tissues were Gram stained. This was in contrast to control mice that died from infection, which exhibited moderate to severe lung lesions and pneumococci could be observed in both the blood and along the surface of the lung tissue. While requiring further investigation before any definitive conclusion can be reached, given the relative delay in the time to death in control mice and the relative lack of lung lesions/colonization in SCD mice, it appears that SCD mice may experience IPD earlier in infection and do not require lung colonization for this to occur, whereas control mice do. Such an explanation would be in line with the *in vivo* bioimaging data presented by Miller et al. [20], which showed that pneumococci remain in the lungs of WT mice 24 hours after infection, but IPD occurs within this same time period in SCD mice.

Given our finding of the overall lack of a difference between the groups in terms of susceptibility infection, we were further surprised when the SCD mice differentially succumbed to infectious challenge after vaccination. It is important to note that the challenge dose after vaccination that we used is two logs higher than the dose used during the infection study (which killed more than half of the mice in both groups). Thus, mice would likely require a protective immune response to be induced from vaccination in order to survive this high challenge dose, and protective antibody responses were only maintained in the control mice at the time of challenge. These findings correlated with an *in vitro* opsonophagocytic killing assay conducted using serum from control and SCD mice 13 weeks after their second booster shot with Prevnar-13, which showed that control mice maintain functional antibody titers, but SCD mice do not. This data has clinical relevance as a recent study in children with SCD demonstrated their inability to sustain anti-PPS3 pneumococcal responses one year after Prevnar-13 vaccination, thus potentially leaving vaccinated children susceptible to infection [21]. This lack of vaccine “take” has important clinical implications and warrants further investigation into the cellular and molecular mechanisms driving this failure.

It has been observed that the administration of antibiotics enhances the deposition of complement components on the surface of *S*. *pneumoniae*, thereby enhancing protective phagocytic responses in conjunction with specific anti-pneumococcal antibodies [22, 23, 24]. It has also been previously shown in SCD patients that have reduced opsonization due to defects in the alternative complement pathway, which acts in conjunction with impaired anti-pneumococcal antibody responses to further inhibit opsonization [25]. It is therefore possible that the co-administration of penicillin and pneumococcal vaccination may be acting synergistically to improve vaccine efficacy in people with SCD. We measured the plasma levels of the complement protein C3 in control and SCD mice, but both groups had normal C3 levels and were not statistically different from each other (data not shown). However, it may still be possible to increase pneumococcal vaccine efficacy in the SCD mouse model through the co-administration of penicillin with Prevnar-13 vaccination.

One potential culprit for lack of protection after vaccination and infectious challenge observed in our model is the well-documented splenic dysfunction and loss of memory IgM/B-1 B-cells found in SCD (which are important for generating anti-polysaccharide antibodies). We have previously shown that these important cells are reduced in proportion in the spleens of SCD mice when compared to controls and correlate with reduced baseline levels of total serum IgM and subclasses of IgG [13]. In this study, we found that serum anti-PPS3 IgG3 titers waned after vaccination, and this subclass of IgG is associated with responses to polysaccharide antigens and is protective against *S*. *pneumoniae* infection [26]. Additionally, antibody titers increased in SCD mice after initial vaccination, but then waned after subsequent booster shots and did not recover. These findings indicate that responses from short-lived plasma cells remain intact in SCD mice; however, long-lived plasma cells do not appear to be generated and/or sustained. Plasma cell longevity has been associated with up-regulation of anti-apoptotic genes [27], suggesting these as intriguing targets for further study in B-cells from people and mice with SCD. Other molecules involved in the long-term differentiation and survival of plasma cells (such as APRIL or Blimp-1) would be worth investigating as well [28, 29]. We should also note that Carter et al. [30] found that using pneumococcal proteins as vaccine antigens also did not confer protection to SCD mice upon infectious challenge (whereas some of the control mice survive). This raises the possibility that SCD mice have immunological impairments in addition to B-1 cell deficiencies.

In addition to the lack of generation of long-lived plasma cells in young SCD mice after pneumococcal vaccination, the absence of a booster response indicates that B-cell memory is also not generated. If such a lack of B-cell memory induction is also observed in SCD patients, this would warrant the need for more frequent vaccination to ensure that protective antibodies are maintained. However, the safety of more frequent vaccination has never been tested in children with SCD and should be approached used with caution. Indeed, we have previously found that vaccination may yield adverse effects in SCD mice [18]. Furthermore, the phenomenon of B-cell exhaustion has recently been described [31], and it is possible that increasing the frequency in which this population is vaccinated could further contribute to the problem.

Pneumococcal infection is an enormous problem in children with SCD, and the utilization of prophylactic strategies to prevent infection is essential to prolong the duration and quality of life in this population. Disturbingly, there have been reports that pneumococcal vaccines may not be as efficacious in children with SCD when compared to the general population. We utilized a SCD mouse model of pneumococcal vaccination and infection to study this problem and found that the polysaccharide-conjugate vaccine Prevnar-13 actually has very poor efficacy in these mice. The reasons for incomplete protection from infection appear to correlate with their inability to maintain polysaccharide-specific antibody titers. The potential candidates for this problem are a lack of the generation and/or maintenance of long-lived plasma cells and/or memory B-cells (likely of B-1 origin). Understanding which correlates of protective immunity are lacking in SCD mice will be essential so that targeted therapeutics can be developed to allow for achievement of long-term immunity after vaccination and subsequent reduction of morbidity and mortality in children with SCD.

## Supporting Information

S1 Text Raw data in S1 Text. (XLSX)
